# Risk Factors for Complicated Lymphadenitis Caused by Nontuberculous Mycobacteria in Children

**DOI:** 10.3201/eid2603.191388

**Published:** 2020-03

**Authors:** Martin Kuntz, Daniela S. Kohlfürst, Cornelia Feiterna-Sperling, Renate Krüger, Ulrich Baumann, Laura Buchtala, Roland Elling, Veit Grote, Johannes Hübner, Markus Hufnagel, Petra Kaiser-Labusch, Johannes Liese, Eva-Maria Otto, Markus A. Rose, Christian Schneider, Volker Schuster, Maximilian Seidl, Olaf Sommerburg, Markus Vogel, Horst von Bernuth, Michael Weiß, Theodor Zimmermann, Alexandra Nieters, Werner Zenz, Philipp Henneke, for the NTMkids Consortium

**Affiliations:** University of Freiburg, Freiburg, Germany (M. Kuntz, R. Elling, M. Hufnagel, C. Schneider, M. Seidl, A. Nieters, P. Henneke);; Medical University of Graz, Graz, Austria (D.S. Kohlfürst, W. Zenz);; Charité–Universitätsmedizin Berlin, Berlin, Germany (C. Feiterna-Sperling, R. Krüger, H. von Bernuth);; Hannover Medical School, Hannover, Germany (U. Baumann);; Professor-Hess-Kinderklinik, Bremen, Germany (L. Buchtala, P. Kaiser-Labusch);; Dr. von Hauner Children's Hospital, Munich, Germany (V. Grote, J. Hübner);; University Children’s Hospital, Würzburg, Germany (J. Liese);; Children’s Hospital, Cologne, Germany (E.-M. Otto, M. Weiß);; Goethe University of Frankfurt, Frankfurt, Germany (M.A. Rose);; University of Leipzig, Leipzig, Germany (V. Schuster);; University of Heidelberg, Heidelberg, Germany (O. Sommerburg);; Heinrich Heine University, Düsseldorf, Germany (M. Vogel);; Friedrich-Alexander-Universität Erlangen-Nürnberg, Erlangen, Germany (T. Zimmermann)

**Keywords:** Nontuberculous mycobacteria, lymphadenitis, epidemiology, risk factors, bacteria, Germany, Austria, tuberculosis and other mycobacteria, children

## Abstract

Nontuberculous mycobacteria (NTM) are an emerging cause of infections, including chronic lymphadenitis in children. To identify risk factors for NTM lymphadenitis, particularly complicated disease, we collected epidemiologic, clinical, and microbiological data on 138 cases of NTM lymphadenitis in children across 13 centers in Germany and Austria. We assessed lifestyle factors but did not identify specific risk behaviors. We noted that more cases of NTM lymphadenitis occurred during cold months than during warm months. Moreover, we noted female sex and age <5.5 years as potential risk factors. Complete extirpation of the affected lymph node appeared to be the best therapeutic measure. We integrated the study data to develop a simple risk score to predict unfavorable clinical outcomes for NTM lymphadenitis.

Nontuberculous mycobacteria (NTM) are common in the environment. NTM frequently are found in soil and are the most common bacteria on showerhead surfaces (*1*,*2*). Although many children likely have daily exposure to NTM, symptomatic infections are rare. Among children <2.5 years of age, the most frequently affected group, the annual NTM incidence in Germany has been estimated to be 3.1 cases/100,000 population (*3*). Some authors suggest the incidence of NTM infections in immunocompetent persons has been increasing in recent years (*4*–*7*), but little longitudinal data in well-defined epidemiologic contexts have been reported.

In children <5 years of age, NTM infections usually manifest as localized cervical lymphadenitis, and many resolve spontaneously. However, the median time to resolution is 40 weeks, differential diagnosis can be challenging, and recurrence and scarring are frequent complications (*8*). NTM infections are hallmarks of several immunodeficiency disorders, especially those involving the interleukin 12 and interferon-γ pathways (*9*–*11*). However, cervical lymphadenitis caused by NTM usually occurs in otherwise immunocompetent children who are not reported to be prone to opportunistic infections later in life. Host and environmental factors that could predispose a child to NTM lymphadenitis remain unclear. To identify potential risk factors for NTM lymphadenitis, we performed a prospective evaluation of childhood NTM lymphadenitis cases across 13 centers in Germany and Austria during 2010–2016. We collected detailed clinical information from the study centers and documented socioeconomic features by using parent-directed questionnaires.

## Materials and Methods

During 2010–2016, the 13 participating centers enrolled all patients <18 years of age who were evaluated for NTM lymphadenitis into the study registry. Most patients were recruited prospectively, but 1 center in Graz, Austria, recruited patients retrospectively from a local registry containing comprehensive data on NTM cases dating back to 2001. In addition to NTM cases, we enrolled a control cohort of patients without chronic illnesses (i.e., with infectious, malignant, or immunologic diseases) who were treated in the same institutions. We matched NTM cases with controls for age, sex, and center.

For comparison with the general population of Germany, we used the nearest neighbor method to match the age and sex of the control cohort with 17,641 participants in the German Health Interview and Examination Survey for Children and Adolescents (KiGGS), 2003–2006 (*12*,*13*). We used 7-fold oversampling to achieve the most accurate matches, resulting in an age- and sex-matched healthy control cohort of 966 children. We compared data on body mass, breast-feeding history, allergies, number of siblings in the same household, smoking during pregnancy, and parents’ education level between the KiGGS-derived and the NTM cohorts.

We considered NTM likely for patients with the following symptoms: cervical lymphadenitis for >3 weeks; a lymph node size of >2 cm; exclusion of other causes, such as bartonellosis, toxoplasmosis, infectious mononucleosis, and lymphoma; and a positive tuberculin skin test (cutoff of 5 mm). We considered NTM to be the definitive diagnosis in patients with typical histology, such as presence of granuloma with or without necrosis or positive Ziehl-Neelsen staining or positive culture or PCR from lymph node samples. We documented the course of disease, including all diagnostic and therapeutic measures, antimicrobial drug therapy, associated clinical problems, prior medical history, vaccination status, and underlying conditions. Parents provided sociodemographic data, including ethnicity, place of birth, duration of breast-feeding, number of patient siblings, daycare attendance, cigarette smoke exposure, and parents’ education and employment, as well as factors potentially increasing NTM exposure, such as animal contact, exposure to water and soil sources, and travel. We tracked patients until they recovered completely. When symptoms remained at the last documented visit, we conducted telephone interviews to assess the remainder of the disease course.

We obtained parental informed consent for all children included in this study. Each participating center’s ethics committee granted ethics approval. The institutional review board of the University Medical Center, University of Freiburg, Freiburg, Germany, was the lead approval agency under IRB no. 232/10. All data were anonymized before analysis.

We performed data analysis by using R 3.5.3 (*14*). We used univariate analysis to screen for potential associations of individual disease history, socioeconomic factors, potential exposure to mycobacteria, and the clinical course of NTM and filtered for a correlation coefficient >0.2 and p<0.05 by using Pearson or Spearman correlations, depending on the variables. We subsequently examined the results for biological plausibility. We compared data from various categories by using the Fisher exact test and compared continuous and ordinal variables by using the Wilcoxon rank-sum test or Welch *t*-test. We analyzed seasonality of NTM by fitting a generalized linear regression model assuming a sinusoid Poisson distribution over the year (cosinor). Using no seasonal pattern as the null hypothesis, we assessed significance of seasonal variance by using the cosinor test (*15*). We considered p<0.05 statistically significant. For some analyses, we combined centers with <20 patients into a single group to attain meaningful comparisons and to ensure appropriate case anonymization.

## Results

### Cohort

During 2010–2016, we recruited 138 patients; 29 were from Graz, Austria, and the rest from Germany, including 35 from Berlin; 27 from Freiburg; 10 each from Leipzig and Munich; 8 from Bremen; 6 from Cologne; 3 each from Düsseldorf, Erlangen, and Heidelberg; 2 from Würzburg; and 1 each from Frankfurt and Hannover. We found suitable controls for 36 patients; 12 cases (9%) remained probable NTM, according to the case definition in the study protocol. We defined the remaining 102 cases as definite NTM.

**Figure 1:**
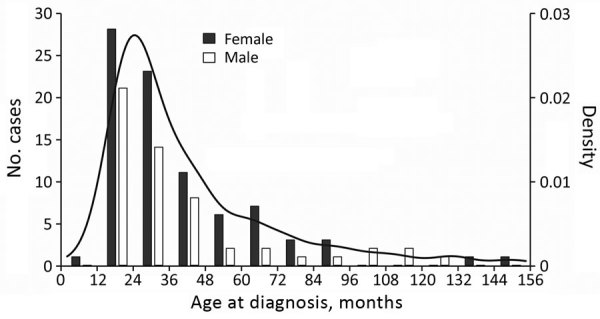
Histogram and density plot of patients’ ages at diagnosis of nontuberculous mycobacteria lymphadenitis across 13 centers in Germany and Austria, 2010–2016.

Most NTM case-patients (61%; p = 0.01) were female. Median age at symptom onset was 28 months (range 8–151 months), with no difference between male and female sex (p = 0.659) (Figure 1). Age and sex distribution were similar between centers (data not shown). Most (86%) patients had parents from Germany or Austria; 14% had parents from a variety of other countries. Most (99.3%) patients were born in Germany or Austria; 1 was born in the Netherlands. Sociodemographic information, history of prior diseases, and behavior related to possible NTM exposure did not differ substantially between female and male patients (data not shown). Boys were reported to play outside in summer longer than girls (p = 0.004); however, the difference was small (median 2 h/d for both sexes and mean 2.1 h/d for boys versus 1.6 h/d for girls). We did not detect any differences between male and female case-patients for any other factors. Of note, none of the patients had received the bacillus Calmette-Guérin vaccine.

### Seasonal Variance

**Figure 2:**
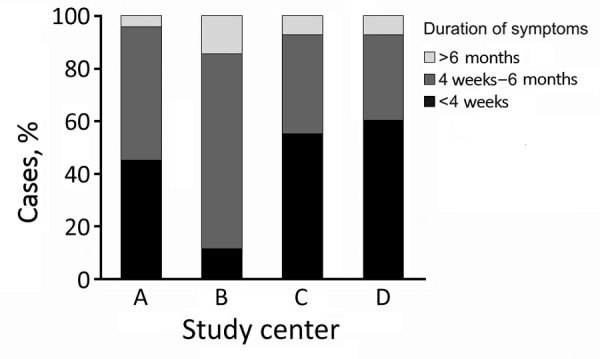
Duration of nontuberculous mycobacteria lymphadenitis symptoms after patients’ first visit to a participating study center across 13 centers in Germany and Austria, 2010–2016. A represents combined data from the 10 smaller centers; B–D represent the 3 largest centers.

**Figure 3:**
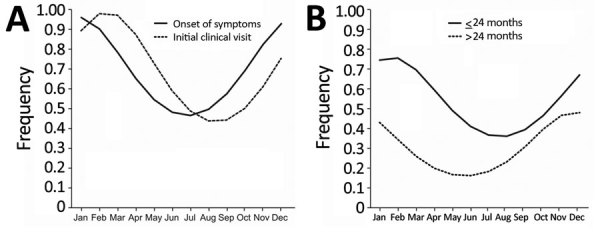
Seasonality of nontuberculous mycobacteria lymphadenitis in children across 13 centers in Germany and Austria, 2010–2016. Curves created by fitting a generalized linear regression model assuming a sinusoid Poisson distribution over the year (cosinor). A) Month of symptom onset and initial visit at a study center. B) Month of symptom onset for patients <24 months and >24 months of age.

We noted more patients sought initial treatment during colder seasons than in warm seasons. Because many patients experienced symptoms long before visiting the clinic, we assessed and documented duration of symptoms at the initial visit. We saw a statistically significant difference between centers for symptom onset and initial visit in the study center (p = 0.019; Figure 2). However, we noted the seasonal pattern for the onset of symptoms across all centers. When we fit a sinusoidal yearly pattern for the reported onset of symptoms, seasonality was statistically significant (p<0.025; Figure 3, panel A). To minimize uncertainty in reporting the duration of symptoms, we analyzed the subgroup of patients with the shortest reported duration of symptoms, <4 weeks. The seasonal pattern remained the same, but statistical significance was lost due to the smaller sample size. In addition, peak incidence differed depending on patients’ ages. For patients <24 months of age, peak incidence occurred in December, but peak incidence occurred 2 months later for older patients (Figure 3, panel B). However, the difference did not reach statistical significance.

### NTM Species

NTM species were reported in 96/138 (70%) cases. In 2 cases, only *M. avium* complex was reported; 9 other cases were listed as NTM unspecified. Cultures were performed on samples from 106 cases; 58% were NTM positive by culture, but NTM species were identified by PCR in 35 culture-negative cases. The longer symptoms lasted before diagnosis, the less likely culture was to be positive. No cases with symptoms that lasted >6 months before diagnosis were culture-positive, compared with 65% of cases that had symptom onset <4 weeks before culture and 57% that had symptom onset in the previous 4 weeks–6 months (p = 0.004). Most (84%) cases were *M. avium* complex, and all centers reported similar rates of *M. avium* (75%–89%). We did not see statistically significant differences in species distribution among centers (p = 0.194; Table 1).

**Table 1:** Number and distribution of patients identified with various *Mycobacteria* species by culture and PCR across 13 centers in Germany and Austria*

*Mycobacteria* species	Total no.	Negative culture, no.	Participating center			
			A	B	C	D
*M. avium*	73	18	29	14	15	15
*M. intracellulare*	6	2	2	2	2	0
MAI complex, nonspecified	2	0	0	0	1	1
*M. malmoense*	4	3	1	0	3	0
*M. kansasii*	4	2	2	0	1	1
*M. haemophilum*	2	2	0	0	2	0
*M. austroamericanum*	1	1	0	1	0	0
*M. bohemicum*	1	0	0	0	0	1
*M. celatum*	1	0	0	1	0	0
*M. gordunae*	1	0	0	1	0	0
*M. stomatepiae*	1	0	1	0	0	0

*Culture remained negative in some cases and nontuberculous mycobacteria were identified only by PCR. Combined data from the 10 smaller centers are represented in A and the 3 largest centers are represented by B, C, and D. MAI, mycobacterium avium-intracellular infection.

### Localization and Symptoms

In most cases, cervical (33%), submandibular (35%), and preauricular (6%) lymph nodes were affected. Only 5% of cases involved lymph nodes in other regions, such as inguinal. Of note, 21% of cases had >1 affected region, such as same-sided cervical and submandibular lymph nodes; 9% of cases had bilateral involvement, mainly occurring as cervical and nuchal localization. We also noted additional local symptoms, such as discoloration, in 59% of cases. Only 17% of cases reported systemic symptoms; most were unspecific, and fever was reported most frequently.

### Diagnosis and Treatment

During the initial workup, most (97%) patients had an ultrasound; 32% had magnetic resonance imaging (MRI), but MRI use was highly variable between centers (range 11%–78%; p<0.001); and 59% (43%–80%; p = 0.005) of patients had a chest radiograph. Tuberculin skin testing was done in 65% (49%–100%; p<0.001) of cases, and 61% of tests were positive. Most (94%) patients received surgical treatment; 49% (32%–69%; p = 0.03) had complete extirpation of the affected lymph node, and 40% had >1 operation, mainly due to impaired wound healing (Table 2). Histologic characteristics of affected lymph nodes included necrosis (61%), granuloma (60%), and giant cells (43%), among other findings (Table 3).

**Table 2:** Patient characteristics, treatment, and outcome for cases of complicated lymphadenitis caused by nontuberculous mycobacteria across 13 centers in Germany and Austria*

Characteristics	Total	Participating center				p value†
		A	B	C	D	
Patient sex, no. (%)	138	47	35	27	29	NS
F	84 (60.9)	29 (61.7)	21 (60)	15 (55.6)	18 (62.1)	NS
M	54 (39.1)	18 (38.3)	14 (40)	12 (44.4)	11 (37.9)	NS
Median patient age, mo	28	28	27	27	37	NS
Treatment, %						
Surgery	94	100	91	89	92	NS
Complete extirpation	49	48	69	42	32	0.03
Antimycobacterial therapy	44	62	21	30	55	<0.001
Outcome, %						
Unfavorable	65	41	68	74	90	0.006
Impaired wound healing	25	19	17	37	34	NS
Facial nerve palsy	7	2	6	0	24	0.002
Mean risk score‡	2.3	1.9	2.6	2.8	2.3	0.004

*Combined data from 10 smaller centers are represented in A and the 3 largest centers are represented by B, C, and D. NS, not significant. †p values are based on original count data and calculated by using the Fisher exact test. ‡Risk scores calculated by using the Kruskal-Wallis test.

**Table 3:** Histologic characteristics of lymph nodes from children infected with nontuberculous mycobacteria across 13 centers in Germany and Austria*

Finding	All samples, %
Fibrosis	6
Necrosis	61
Granulomatous infiltration	19
Granuloma	60
Giant cells	43
Epithelial cells	18
Acid-fast bacilli	15

*Multiple findings possible.

Apart from surgery, treatment varied considerably among centers (Table 2). Only 34% of patients received appropriate mycobacteria-targeted antimicrobial therapy that included a macrolide for >3 months (10%–52% across centers; p<0.001) (*16*,*17*). Only 23% of patients with complete extirpation of the affected lymph node received appropriate antimycobacterial therapy, compared with 49% undergoing other types of surgery (p = 0.006). The percentage of patients receiving antimycobacterial therapy declined over the course of the study (data not shown).

### Clinical Course and Outcomes

We used univariate and multivariate analyses to search for risk factors associated with surgical complications, relapse after surgery, and length of time to full recovery. In addition to single factors, we created a surrogate, “unfavorable outcome,” which we defined as illness lasting >12 months, >1 surgical intervention at the same site, or occurrence of major complications, such as substantial scarring or facial nerve palsy. We found 65% of the cohort had an unfavorable outcome and that differences between centers were statistically significant, ranging from 41% to 90% (p<0.01). However, we did not note any statistically significant difference between the 3 largest centers, where unfavorable outcomes averaged 78% (p = 0.19). The difference in the rate of transient or persistent facial nerve palsy was statistically significant between centers, ranging up to 24% (p<0.01). The incidence of facial nerve palsy did not correlate with the size of the study center, which we measured by the number of cases included from a center (Table 2).

The factor most strongly associated with unfavorable outcome was the presence of liquefaction in the affected lymph node identified by ultrasound or MRI. Most (73%) patients with liquefaction had an unfavorable outcome, compared with only 40% of patients who did not (p = 0.009).

Surgical procedures also affected outcomes. Overall, 51% of patients who had complete primary extirpation had an unfavorable outcome, compared with 75% of patients who had other types of surgery (p = 0.028). Only 19% of patients who had primary extirpation had impaired wound healing, compared with 35% of patients who had other types of surgery (p = 0.049). Complete extirpation also was associated with a lower incidence of other complications; only 3% of these patients had facial nerve palsy compared with 12% who had other surgical procedures (Table 3). Of patients who had the affected lymph node drained, 60% experienced impaired wound healing and an unfavorable outcome (p = 0.024), compared with 25% of patients who received other therapies (p = 0.027).

Mycobacteria-targeted antimicrobial therapy was not associated with improved clinical outcomes. When we used multivariate analysis to correct for the influence of the treatment center, complete extirpation of the affected lymph node still predicted a favorable clinical outcome (p = 0.029), good primary wound healing after surgery (p = 0.026), and a low rate of postsurgical complications, such as facial nerve palsy (p = 0.01). We saw impaired wound healing more often in connection with local skin symptoms, such as discoloration (p = 0.027), increased size of the affected lymph node (p = 0.036), and >1 affected location (p = 0.039). Taken together, complete extirpation was associated with an overall favorable clinical outcome and a lower rate of local complications compared with other types of surgical intervention.

**Figure 4:**
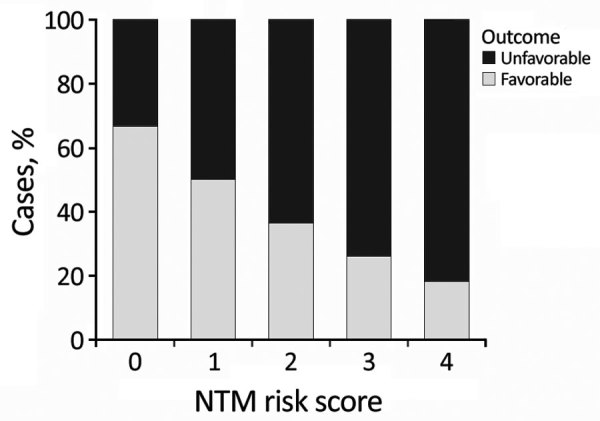
Correlation of NTM risk score and surrogate clinical outcome in a study of NTM lymphadenitis in children across 13 centers in Germany and Austria, 2010–2016. Scores represent 1 point each for skin discoloration, lymph node >2 cm, liquefication of lymph node on ultrasound or magnetic resonance imaging, and >1 affected location. Outcome percentages calculated by Pearson correlation are dependent on the assigned score (r = 0.23, p = 0.036). NTM, nontuberculous mycobacteria

Using these results, we developed a score for estimating the risk for an unfavorable clinical course before surgery. We grouped factors associated with adverse outcomes. Because the sample size was small, we could not calculate effect sizes reliably. Instead, we assigned each of the following items 1 point: skin discoloration, lymph node >2 cm, liquefaction on ultrasound or MRI, and >1 affected location. The resulting score helped us demonstrate a statistically significant association between outcomes and local complications, such as impaired wound healing, which has a Pearson score of *r* = 0.23 (p = 0.036) (Figure 4).

### Risk Factors for NTM Lymphadenitis

To identify candidate risk factors for NTM lymphadenitis, we recruited control patients matched for age, sex, and center. In addition, we selected an age- and sex-matched control cohort from the KiGGS 2003–2006 (*13*). We compared sociodemographic factors, medical history, and data relating to potential exposure to mycobacteria, such as contact with animals and water sources, between patients and controls. Sociodemographic characteristics, such as parents’ level of education, a child’s presence in daycare, household smoking, and number of siblings, did not differ between patients and controls. We saw no difference in height and weight, the rate or duration of breast-feeding (mean 5.7 months for NTM patients and 5.5 months for control cohort; p = 0.590), or adherence to recommended vitamin D supplementation in the first year of life (94% for NTM patients and 81% for control cohort; p = 0.151).

We detected no difference between groups in health-related factors, such as frequency of antimicrobial drug treatment, typical childhood infections, or allergies. We also did not detect any difference in factors related to possible exposure to NTM, such as number of hours playing outside per day, exposure to natural bodies of water or swimming pools, or contact with pets and farm animals.

## Discussion

In this study, we found that girls 18–36 months of age were at highest risk for NTM lymphadenitis, which aligns with findings of studies conducted in other geographic locations (*3*,*4*,*7*,*18*–*21*). The underlying cause for the predominance of NTM in girls remains unclear. We speculate that exposure to soil-residing NTM could be associated with the increased risk for NTM infection. However, the amount of time girls spent outside did not correspond to disease risk. Moreover, the predominance of NTM in female patients in varying geographic and climatic regions suggests that differences in immunity or host–pathogen interaction are more likely to be responsible for sex-related differences in NTM lymphadenitis incidence, rather than differences in exposure to NTM.

We also found a seasonal incidence pattern that peaked during colder months. Other studies reported an increase in new cases during winter (*7*,*20*), but peak incidence in relation to temperature and length of daylight hours varied among studies in different geographic regions. To expand on other studies, we recorded the duration of symptoms at each patient’s initial visit to participating centers. Using this information, we were able to correct for the time between onset of symptoms and diagnosis because differences in referral and availability of appointments might influence the seasonal pattern. We noticed that time from onset of symptoms to the first physical examination at a clinic varied greatly between centers. Year-round differences in NTM exposure also could affect disease incidence. For example, seasonal NTM occurrence in drinking water has been reported (*22*,*23*).

Furthermore, the low incidence of NTM infections in warmer months could be linked to seasonal variations in vitamin D levels. Variations in vitamin D metabolism previously have been linked to NTM susceptibility (*24*,*25*). However, in our study, patients and healthy controls did not differ in sunlight exposure, and vitamin D supplementation in the first year of life occurred more often in the NTM-infected cohort than in controls.

Given the slow replication of NTM, incubation time for NTM lymphadenitis is unknown. Thus, a critical timeframe for infection is difficult to establish from the seasonal pattern seen in our cohort. In addition, unknown host factors, such as concurring infections, might affect seasonal incidence.

*M. avium* causes the majority of NTM lymphadenitis cases in countries as far removed from each other as Germany, Sweden, and Australia (*3*,*4*,*7*,*18*). By contrast, NTM species isolated in pulmonary infections have much more pronounced regional differences (*26*). Other than *M. avium*, we noted variations in NTM species between centers, but the small number of cases precluded a more detailed analysis. Nevertheless, the relatively high number of rare species underlines the need for improved PCR and culture techniques for reliable diagnosis of uncommon NTM species.

Therapeutic procedures, disease outcomes, and complications varied substantially among centers and over the course of the study. Overall, patients frequently had complicated clinical courses that lasted >12 months or major complications, even after complete extirpation, the treatment most associated with a favorable outcome. More than 75% of patients in the 3 most experienced study centers had complicated clinical courses, which is a striking contrast to a report by Lindeboom et al. (*16*) in which early complete lymph node extirpation cured >90% of lymphadenitis cases. However, lymphadenitis frequently is caused by factors other than NTM. Our data might reflect more on clinical practices in which early extirpation is more the exception than the rule, a hypothesis further supported by the fact that clinical outcomes did not correlate with center size in our study. Consequently, surgical experience might not explain the unfavorable outcomes in our study. However, because only specialized centers participated in the study and inclusion criteria were strict, complicated surgical cases probably were overrepresented in our cohort. This finding is supported by evidence that some patients underwent primary surgery elsewhere and were referred to our participating centers after they received a definitive NTM diagnosis.

The rate of facial nerve palsy in our study was within the range reported elsewhere (*7*), but we saw variations among centers. Because our study relied on local clinical data, we cannot rule out differences in sensitivity thresholds. However, differences in the surgical approach reflected by the varying rates of complete extirpation most likely explain this observation. Primary complete lymph node extirpation was associated with a lower rate of facial nerve palsy than other surgical procedures. However, this observation could have a high bias because complete extirpation is performed more frequently when the affected lymph node is farther from the facial nerve. Antimicrobial therapy for NTM lymphadenitis decreased over the course of the study, which might reflect the increasing number of studies questioning the therapeutic benefit of antimicrobial drugs to treat NTM lymphadenitis.

Our study has several limitations. First, the limited number of disease controls precluded a more detailed analysis of possible individual risk factors. Second, our cohort probably does not reflect the full variety of NTM lymphadenitis phenotypes. Our strict inclusion criteria and registry-like design likely overrepresented severe cases, which are seen more frequently in secondary and tertiary health centers, and underrepresented less severe cases. Third, because we relied on data acquired by local centers and did not collect detailed information on PCR methods, we cannot rule out differences in laboratory methods or interpretation of clinical findings.

In conclusion, the NTM lymphadenitis risk profile for female patients <5 years of age, the chronic (albeit usually benign) course of disease, the worldwide predominance of *M. avium*, and the seasonal variability we noted in our study suggest a complex contribution of host, pathogen, environmental, and potential microbiome factors. Individual factors are insufficient to grasp the risk for unfavorable clinical outcomes. Our proposed risk score comprises multiple items and could be useful in estimating NTM lymphadenitis risk and stratifying patients to therapeutic modalities, if its validity is confirmed in a prospective study. Until then, early and complete extirpation of a suspicious lymph node remains a mainstay of NTM diagnosis and therapy.
